# Supplementary figures and images for: Genotype-dependent response to water deficit: increases in maize cell wall digestibility occurs through reducing both p-coumaric acid and lignification of the rind
Source: Front Plant Sci. 2025 May 9;16:1571407. doi: 10.3389/fpls.2025.1571407 (PMC12098441; doi:10.3389/fpls.2025.1571407)

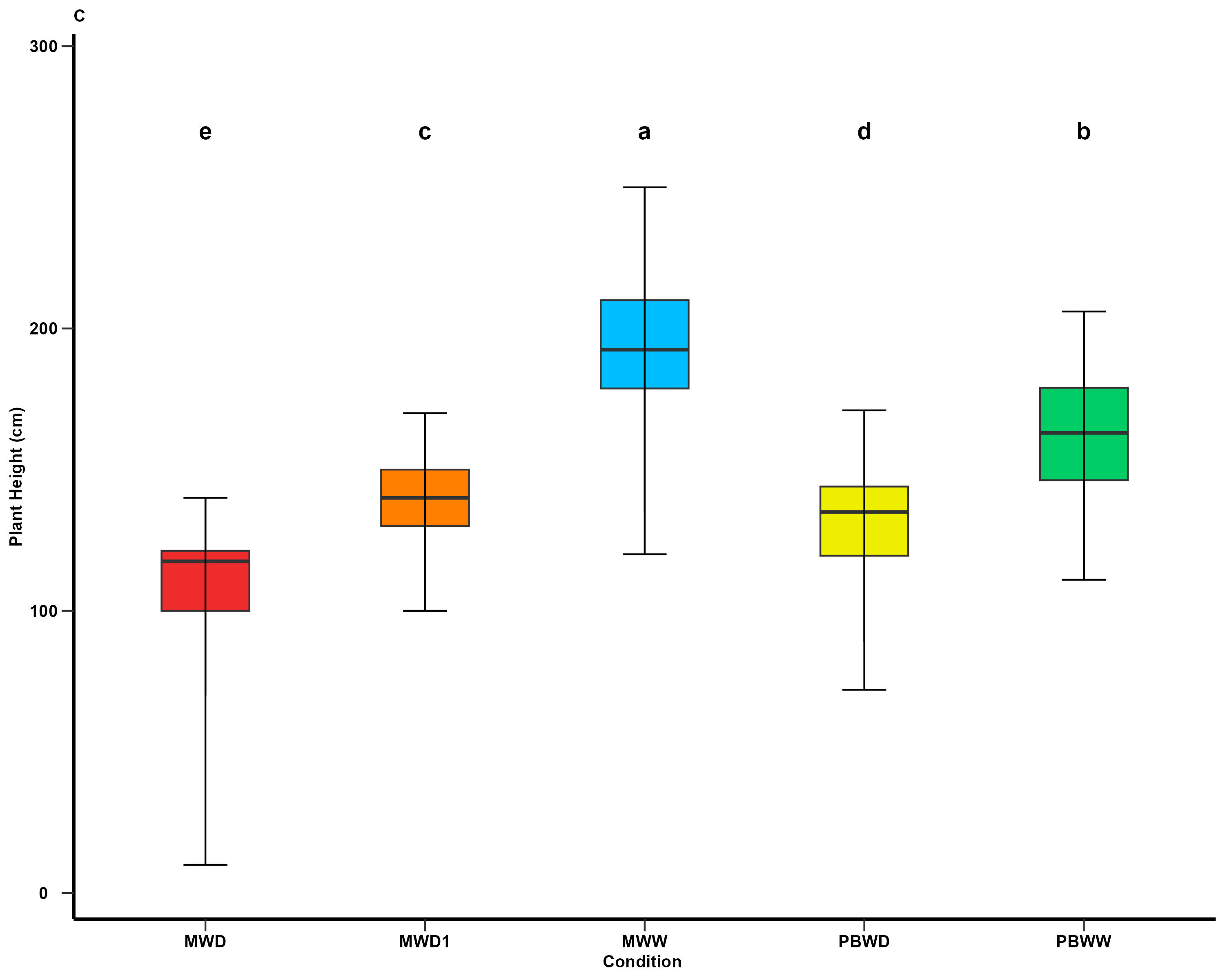

Supplement: Supplementary Figure 1 — Means comparison of Plant Height under different irrigation treatments. Different letters indicate significant differences between conditions (p < 0.05). [file Image1.jpeg]

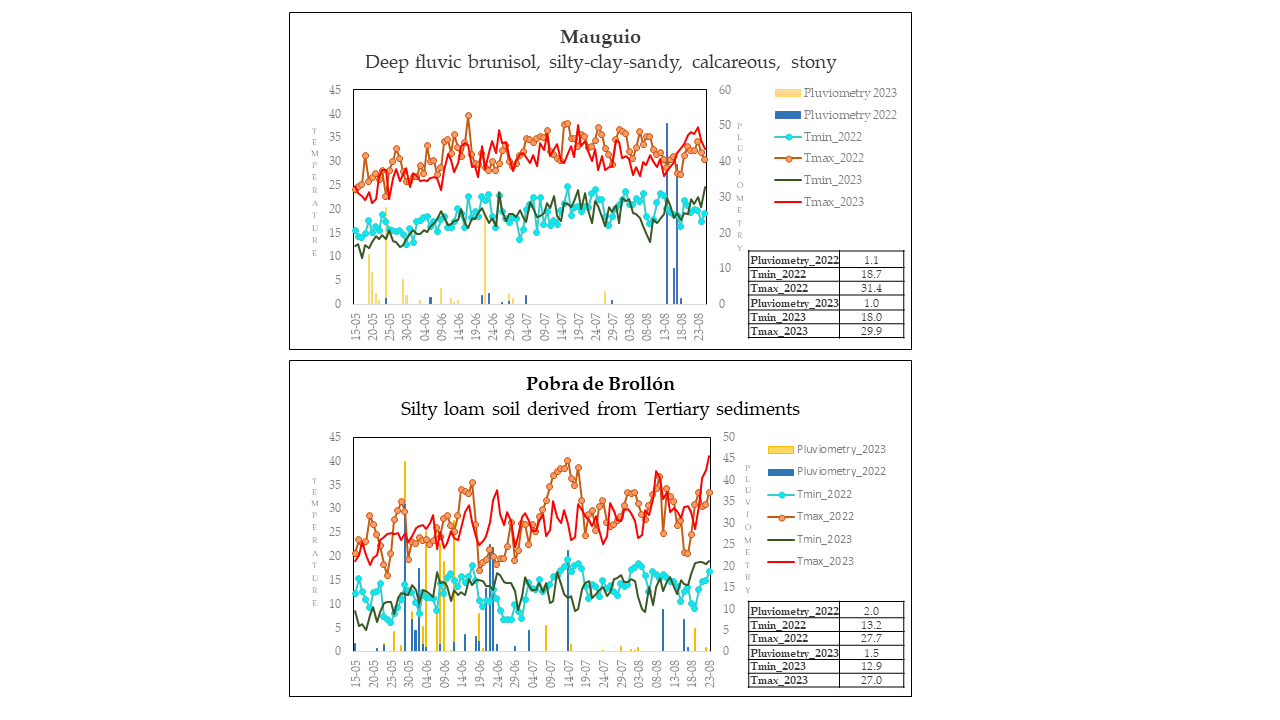

Supplement: Supplementary Figure 2 — Climatic conditions across locations (Pobra de Brollón, Mauguio) and growing seasons: Maximum and minimum temperatures (lines) and accumulated precipitation (bars) for the 2022 and 2023 seasons. [file Image2.tif]
